# Hospitalization rates for complications due to systemic therapy in the United States

**DOI:** 10.1038/s41598-021-86911-x

**Published:** 2021-04-01

**Authors:** Anshul Saxena, Muni Rubens, Venkataraghavan Ramamoorthy, Raees Tonse, Emir Veledar, Peter McGranaghan, Subrina Sundil, Michael D. Chuong, Matthew D. Hall, Yazmin Odia, Minesh P. Mehta, Rupesh Kotecha

**Affiliations:** 1 Baptist Health South Florida, Miami, FL USA; 2 Florida International University, Miami, FL USA; 3 Department of Radiation Oncology, Miami Cancer Institute, Baptist Health South Florida, Miami, FL USA; 4 University of Central Missouri, Warrensburg, MO USA; 5 Southeastern Regional Medical Center, Lumberton, NC USA

**Keywords:** Cancer, Oncology

## Abstract

The aim of this study was to estimate the trends and burdens associated with systemic therapy-related hospitalizations, using nationally representative data. National Inpatient Sample data from 2005 to 2016 was used to identify systemic therapy-related complications using ICD-9 and ICD-10 external causes-of-injury codes. The primary outcome was hospitalization rates, while secondary outcomes were cost and in-hospital mortality. Overall, there were 443,222,223 hospitalizations during the study period, of which 2,419,722 were due to complications of systemic therapy. The average annual percentage change of these hospitalizations was 8.1%, compared to − 0.5% for general hospitalizations. The three most common causes for hospitalization were anemia (12.8%), neutropenia (10.8%), and sepsis (7.8%). Hospitalization rates had the highest relative increases for sepsis (1.9-fold) and acute kidney injury (1.6-fold), and the highest relative decrease for dehydration (0.21-fold) and fever of unknown origin (0.35-fold). Complications with the highest total charges were anemia ($4.6 billion), neutropenia ($3.0 billion), and sepsis ($2.5 billion). The leading causes of in-hospital mortality associated with systemic therapy were sepsis (15.8%), pneumonia (7.6%), and acute kidney injury (7.0%). Promoting initiatives such as rule OP-35, improving access to and providing coordinated care, developing systems leading to early identification and management of symptoms, and expanding urgent care access, can decrease these hospitalizations and the burden they carry on the healthcare system.

## Introduction

Cancer continues to be a leading cause of morbidity and mortality, and currently an estimated 15.5 million cancer survivors are living in the United States^1^. Cancer treatments have significantly improved survival rates and quality of life, leading to an increasing number of patients receiving ongoing treatment^2,3^. In fact, every year in the United States, an estimated 650,000 patients receive systemic therapy as part of their cancer treatment^4^. Although systemic therapies have led to improved survival rates overall, they have also been associated with adverse events, some of which may result in hospitalization^5,6^. Many studies have described hospitalizations among cancer patients receiving systemic therapy^5,7–11^. The majority of these studies show that complications such as neutropenia, thrombocytopenia, anemia, infections, fever, mucositis, dehydration, and nausea and vomiting are responsible for the majority of hospitalizations.

Hospitalizations due to complications of systemic therapy involve significant expenditures and can be a significant burden for both cancer patients and the healthcare system. A report published in 2007 by MedStat showed that each such hospitalization incurred an average expenditure of $22,000 per patient^12^. In addition, hospitalizations for complications can result in treatment interruptions and adversely affect patients’ responses to treatment. Understanding the characteristics of these hospitalizations could help physicians and other healthcare providers to develop preventive strategies and manage complications in outpatient settings. To the best of our knowledge, there is no study that has characterized hospitalizations for complications of systemic therapy at the national level. Therefore, this study sought to identify the trends as well as the burdens associated with these hospitalizations using a nationally representative dataset.

## Methods

### Data source

We used National Inpatient Sample (NIS) data from 2005 to 2016 to characterize hospitalizations for complications of systemic therapy. NIS is the largest all-payer inpatient database in the United States and was developed by the Agency for Healthcare Research and Quality (AHRQ) as a part of Healthcare Cost and Utilization Project (HCUP)^13^. NIS has enabled researchers and policymakers to estimate parameters such as healthcare utilization and outcomes, hospitalization cost, access and quality of care, and total healthcare spending at the national level, which are useful for healthcare policy decisions. Each year, the NIS collects data from more than 7 million hospitalizations in the United Sates, which corresponds to 35 million weighted hospitalizations. To increase its ability in capturing national estimates, the NIS redesigned its data collection methods in 2012. The NIS currently collects and stores a sample of discharge records from all hospitals participating in HCUP, rather than all discharge records collected from a sample of hospitals (as was its practice prior to 2012). Hospitalization data within the NIS contains one primary and as many as 29 secondary diagnoses, in addition to other variables, such as demographics, hospital data, clinical procedures, length of stay, disposition status, and total charges. *International Classification of Diseases, Ninth Revision, Clinical Modification (ICD-9-CM)* codes and *Tenth Revision (ICD-10-CM)* codes were used for reporting primary and secondary diagnoses in this study.

### Study design

The current study was a retrospective analysis of NIS data collected from 2005 to 2016. Cancer patients were identified through Clinical Classifications Software (CCS) codes 11–45 indicating neoplasms as described previously^14^. Cancers were categorized into solid tumors and hematologic malignancies (Table 1). Complications of systemic therapy were identified using *ICD-9-CM* and *ICD-10-CM* external causes-of-injury codes (E-codes) (Supplement File). The first listed non-cancer diagnosis identified using the *ICD-9-CM* or *ICD-10-CM* diagnosis code as the primary reason for hospitalization (eTable S1). The procedures used for selecting the patient cohort are presented in the CONSORT diagram (eFig. S1). Other variables included demographics (age, sex, race), socioeconomic factors (median household income by zip code, insurance type), and hospital characteristics (region, bed size, and teaching status). Primary outcome variables included hospitalization rate and total cost; secondary outcome variables included hospital length of stay and in-hospital mortality for complications. We followed the Strengthening the Reporting of Observational Studies in Epidemiology (STROBE) guidelines for reporting our findings. The methods for this study were conducted in accordance with relevant guidelines and regulations. We followed the Strengthening the Reporting of Observational Studies in Epidemiology guidelines for reporting our findings.

**Table 1:** Hospitalizations for complications of systemic therapy by tumor type, 2005–2016 (n = 2,419,722).

Tumor type	Number of hospitalizations, n (%, 95% CI)
**Solid**	
Bladder	43,399 (1.8%, 1.6–1.8%)
Bone and connective tissue	81,465 (3.3%, 3.0–3.4%)
Brain and nervous system	40,399 (1.6%, 1.5–1.7%)
Breast	275,956 (11.1%, 10.8–11.3%)
Cervix	27,487 (1.1%, 1.0–1.2%)
Colon	144,433 (5.8%, 5.6–5.9%)
Esophagus	39,130 (1.6%, 1.5–1.8%)
Head and neck	79,670 (3.2%, 3.1–3.3%)
Kidney and renal	45,054 (1.8%, 1.7–1.9%)
Liver and intrahepatic bile duct	28,466 (1.1%, 1.1–1.2%)
Lung	362,761 (14.6%, 14.2–15.0%)
Melanoma	36,696 (1.5%, 1.3–1.6%)
Other	47,057 (1.9%, 1.7–2.0%)
Ovary	87,741 (3.5%, 3.4–3.6%)
Pancreas	73,264 (3.0%, 2.8–3.2%)
Prostate	75,842 (3.1%, 2.9–3.3%)
Rectum and anus	76,460 (3.1%, 3.0–3.2%)
Stomach	39,917 (1.6%, 1.5–1.7%)
Testis	21,050 (0.85%, 0.78–0.91%)
Thyroid	9117 (0.47%, 0.34–0.38%)
Uterus	39,406 (1.6%, 1.5–1.8%)
**Liquid**	
Hodgkin lymphoma	40,677 (1.6%, 1.5–1.7%)
Non-Hodgkin lymphoma	334,229 (13.5%, 13.2–13.7%)
Multiple myeloma	117,879 (4.8%, 4.5–4.9%)
Leukemia	385,258 (15.5%, 15.0–16.0%)

*The total number of hospitalizations for all cancers considered together is be less than the sum of the hospitalizations for individual cancers because some patients had multiple cancers.

The study was reviewed by the Miami Cancer Institute’s Institutional Review Board (IRB), which exempted the study from IRB approval. The IRB also waived the requirement for informed consent because it used previously collected de-identified data stored in NIS.

### Statistical analysis

Statistical analyses were performed using SAS (version 9.4, SAS Institute, Cary, North Carolina), which accounts for the complex survey design and clustering. The guidelines for using NIS data developed by Khera and Krumholz were used to ensure appropriate procedures for the study^15^. As already mentioned, the NIS was redesigned in 2012 to improve national estimates. To account for these changes, we used modified discharge weights for the years 2005–2011^16^. Descriptive statistics were used to understand temporal factors, types of cancers, demographics, socioeconomic factors, and hospital characteristics. Hospitalization rates were calculated by dividing the total number of patients hospitalized due to complications of systemic therapy by the total number of hospitalizations. Costs of individual inpatient-stays were calculated by multiplying total hospital charges by cost-to-charge ratios. The costs for each year were adjusted according to 2016 inflation levels, based on the US Consumer Price Index. Hospital length of stay was estimated by subtracting the admission date from the discharge date. In-hospital mortality was calculated by dividing the number of patients admitted for complications of systemic therapy and those who died in the hospital by the total number of patients hospitalized due to complications of systemic therapy. All analyses included weighted frequencies for calculating national estimates. Statistical significance was set at *P* < 0.05.

### Conference presentation

This study was presented at the ASCO Annual Meeting from May 29–31, 2020.

## Results

There were 443,222,223 weighted hospitalizations recorded during the period 2005–2016, of which 2,419,722 were due to complications of systemic therapy. The majority of patients (55.3%, 95% CI 44.2–45.7%) were ≥ 60 years old and female (52.5%, 95% CI 51.3–51.8%) (eTable S2). The majority of the patients were White (66.2%, 95% CI 65.8–67.9%), followed by Blacks (9.5%, 95% CI 9.1–9.8%), and Hispanics (8.7%, 95% CI 7.7–8.9%). Nearly 65.2% (95% CI 63.7–66.6%) of the patients were admitted in hospitals with large bed capacity, and 63.8% (95% CI 61.2–64.3%) were admitted to urban teaching hospitals. Region-wide distribution showed that the majority of the patients were admitted in the South (36.7%, 95% CI 35.1–38.4%), followed by the Midwest (24.0%, 95% CI 22.4–25.4%), the West (21.1%, 95% CI 19.3–22.0%) and the Northeast (18.2%, 95% CI 17.2–19.9%). About 4.6% (95% CI 4.3–4.7%) of the patients died during hospitalization for complications of systemic therapy. During the study period, the average annual percentage change (AAPC) in hospitalization rates for complications of systemic therapy was 8.1% (95% CI 7.3–9.1%), compared to − 0.53% (95% CI − 1.1% to − 0.21%) for general hospitalizations.

**Figure 1:**
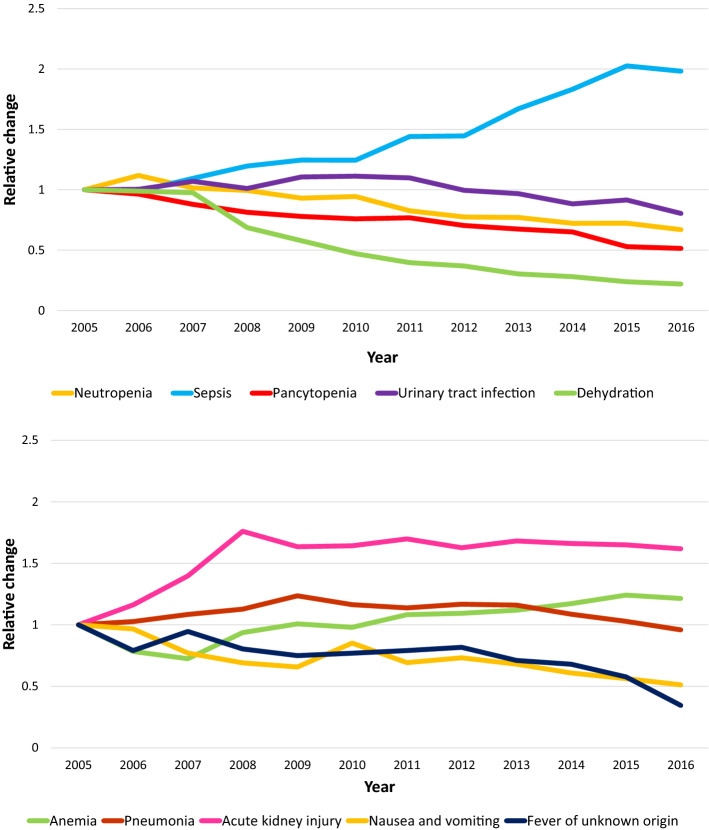
Relative change in the number of hospitalizations for neutropenia, sepsis, pancytopenia, urinary tract infection, dehydration, anemia, pneumonia, acute kidney injury nausea and vomiting, and fever of unknown origin from 2005 to 2016.

During the study period, hospitalizations for complications of systemic therapy had the highest relative increase for sepsis (1.9-fold) and acute kidney injury (1.6-fold), and the lowest relative increase for dehydration (0.21 fold) and fever of unknown origin (0.35-fold) (Fig. 1).

Table 2 shows the top 10 complications associated with systemic therapy in terms of the number of hospitalizations, hospital length of stay, in-hospital mortality, and charges. The 3 most common reasons for hospitalizations were anemia (12.8%, 95% CI 9.7–13.1%), neutropenia (10.8%, 95% CI 7.9–11.0%), and sepsis (7.8%, 95% CI 6.0–8.1%). The length of stay was highest for sepsis (median, 6.1 days, 3.4–11.5), pneumonia (5.5 days, 3.1–10.4), and acute kidney injury (5.2 days, 2.8–10.2). The leading causes of in-hospital mortality included sepsis (15.8%, 15.4–17.9%), pneumonia (7.6%, 95% CI 7.1–10.6%), and acute kidney injury (7.0%, 95% CI 6.6–10.2%). Costliest complications were sepsis (median, $16,834, 95% CI $16,361-$17,307), acute kidney injury ($13,172, 95% CI $12,381-$13,963), and pneumonia ($13,040, 95% CI $12,541-$13,538).

**Table 2:** Number of hospitalizations, hospital length of stay, in-hospital mortality, and charges for the top 10 complications of systemic therapy, 2005–2016 (n = 2,419,722).

Diagnosis	Number of hospitalizations (%, 95% CI)	Length of stay in days, median (IQR)	Mortality, % (95% CI)	Median Charges per Hospitalization (95% CI)	Total Charges in Billions (95% CI)
Anemia	309,724 (12.8%, 9.7–13.1%)	4.9 (2.4–14.6)	2.8% (2.6–3.4%)	$12,024 ($11,373–$12,674)	$4.62 ($4.11–$5.12)
Neutropenia	261,330 (10.8%, 7.9–11.0%)	3.9 (2.3–6.5)	1.6% (1.4–2.8%)	$9091 ($8804–$9377)	$2.49 ($2.24–$2.74)
Sepsis	188,738 (7.8%, 6.0–8.1%)	6.1 (3.4–11.5)	15.8% (15.4–17.9%)	$16,834 ($16,361–$17,307)	$3.01 ($2.78–$3.22)
Pneumonia	106,468 (4.4%, 3.4–4.8%)	5.5 (3.1–10.4)	7.6% (7.1–10.6%)	$13,040 ($12,541–$13,538)	$1.65 ($1.49–$1.82)
Acute kidney injury	87,110 (3.6%, 2.8–3.7%)	5.2 (2.8–10.2)	7.0% (6.6–10.2%)	$13,172 ($12,381–$13,963)	$1.34 ($1.17–$1.51)
Nausea with vomiting	77,431 (3.2%, 2.3–3.3%)	2.6 (1.4–4.4)	0.70% (0.55–1.1%)	$6059 ($5856–$6261)	$0.43 ($0.40–$0.47)
Dehydration	72,592 (3.0%, 2.0–3.4%)	3.0 (1.6–5.5)	2.8% (2.5–3.6%)	$6351 ($6159–$6543)	$0.51 ($0.47–$0.55)
Urinary tract infection	31,459 (1.3%, 0.93–1.4%)	3.6 (2.1–6.2)	1.5% (1.1–4.4%)	$8460 ($8135–$8784)	$0.29 ($0.26–$0.32)
Congestive heart failure	31,456 (1.3%, 0.87–1.5%)	4.0 (2.3–7.1)	4.1% (3.6–5.1%)	$10,120 ($9701–$10,537)	$0.32 ($0.29–$0.35)
Fever of unknown origin	26,617 (1.1%, 0.71–1.2%)	2.4 (1.4–4.2)	0.33% (0.18–1.1%)	$6236 ($5920–$6552)	$0.18 ($0.15–$0.21)

**Figure 2:**
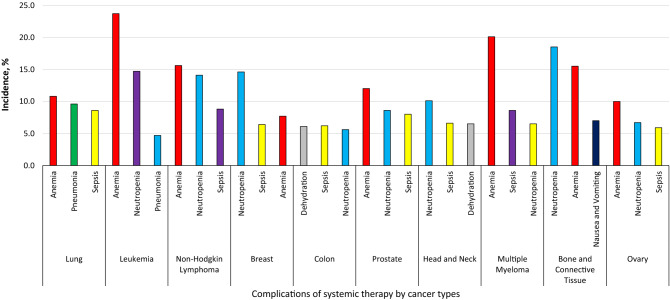
The most common complications of systemic therapy by the top 10 most common cancer types.

Subsequently, complications of systemic therapy were analyzed by cancer type, and the most common cancers were leukemia (15.5%, 95% CI 13.6–16.1%), lung cancer (14.6%, 95% CI 13.1–15.9%), and non-Hodgkin lymphoma (13.5%, 95% CI 12.2–16.1%). The most common complications of systemic therapy among solid tumors were neutropenia (9.4%, 95% CI 8.5–10.8%), anemia (9.2%, 95% CI 7.2–11.4%), and sepsis (7.3%, 95% CI 6.3–9.9%), and among hematologic malignancies were anemia (19.7%, 95% CI 17.6–21.3%), neutropenia (13.4%, 95% CI 11.2–15.9%), and sepsis (8.8%, 95% CI 6.5–10.5%). Although anemia, neutropenia, and sepsis were among the top 3 complications of systemic therapy in the majority of cancer types, exceptions included bone and connective tissue cancers, where nausea and vomiting were among the top 3 complications, and colon and head and neck cancers, where dehydration was among the top 3 complications (Fig. 2). Non-Hodgkin lymphoma (13.1%, 95% CI 10.4–16.2%) and leukemia (10.4%, 95% CI 8.1–12.7%) were the most common malignant neoplasms among patients with acute kidney injury, and breast cancer (22.1%, 95% CI 19.9–24.5%) and non-Hodgkin lymphoma (20.3%, 95% CI 18.5–23.1%) were the most common malignant neoplasms among patients with congestive heart failure.

## Discussion

To the best of our knowledge, this is the first study that reports on hospitalizations for complications of systemic therapy, using a nationally representative dataset. In this study, hospitalizations for complications of systemic therapy increased significantly, in contrast to a decrease in general hospitalizations for all causes during the same study period. A significant majority of these hospitalizations consisted of older adults greater than 60 years of age on Medicare benefits and admitted to urban teaching centers. Complications associated with systemic therapy that resulted in the highest number of hospitalizations were anemia, neutropenia, and sepsis.

The observed increase in hospitalizations for complications of systemic therapy could be explained by an increase in the number of elderly patients who are becoming eligible for systemic therapy^1^. As newer targeted treatments have significantly improved overall survival and progression-free survival rates in the overall population, these benefits have also been translated to elderly patients as well^17,18^. Nevertheless, these new agents have also increased the number of adverse events because of side effects, in addition to those already resulting from existing treatment regimens^19^. These factors could be responsible for increasing the volume of hospitalization for managing these conditions. The majority of the patients were admitted in large urban teaching hospitals as cancer management requires intensive treatment and follow-up most widely available in higher-tiered hospitals^20^.

Among patients receiving systemic therapy for both solid tumors and hematologic malignancies, anemia, neutropenia, and sepsis were the most common complications requiring hospitalization. This could be attributed to profound myelosuppression and immunosuppression associated with chemotherapy and bone marrow transplantation. In addition, bone marrow transplant recipients are more likely to experience readmissions for infections and graft failures in the initial month after discharge from a primary hospitalization^21^. Nausea and vomiting were common among patients receiving systemic therapy for bone and connective tissue cancers. These cancers are treated with consecutive-day regimens of multiple anti-cancer drugs, resulting in higher levels of nausea and vomiting prominent in cisplatin-containing regimens^22^. Dehydration was common among colon and head and neck cancers. This could be due to complications such as mucositis, common in these patients, limiting their ability to consume food and beverages, as well as destruction of the lining of the gastrointestinal tract, limiting the absorption of fluids and electrolytes^23^. Acute kidney injury was common among Non-Hodgkin lymphoma and leukemia patients. Kidney injury in these conditions could be caused by the malignancy itself or as a side effect of systemic therapy^24^. Congestive heart failure was common among non-Hodgkin lymphoma and breast cancer patients. This finding could be due to the adverse effects of medications such as trastuzumab and anthracycline, commonly recommended for these cancers^25^.

In our study, sepsis, pneumonia, and aspiration pneumonitis were associated with prolonged length of stay and mortality among those receiving systemic therapy. This is not surprising as cancer treatments cause significant immunosuppression and disruption of mucosal barriers. Some studies have reported a significant increase in the length of stay and higher mortality rates when cancer was complicated by severe sepsis^26,27^. Pneumonia and aspiration pneumonitis are usually severe in leukemia and lung cancer patients and hence associated with greater length of stay and mortality^28,29^.

The complications that were associated with the highest total charges were anemia, sepsis, neutropenia, and pneumonia. Though sepsis had the highest median cost, total charges were highest for anemia because of the large volume of patients admitted for this condition. Previous studies evaluating the financial burdens of cancer hospitalization have shown similar findings. For example, in a large-scale study among breast cancer patients, anemia and neutropenia had the highest expenditures^30^. Similarly, in a retrospective study among 412,005 cancer patients of all types, sepsis was among the 3 most costly complications^29^.

Hospitalizations for complications of systemic therapy involve significant financial burdens in addition to poor quality of life^29^. Efforts to lower these expenditures could enormously decrease healthcare costs. An important step in this direction would be to accurately identify unplanned hospitalizations in cancer care. Rule OP-35, proposed by the Centers for Medicare and Medicaid Services (CMS), offers hope in this direction^31^. This rule proposes to identify patients who have received systemic therapy in outpatient settings and subsequently hospitalized for 10 major complications such as anemia, neutropenia, sepsis, pneumonia, nausea, vomiting, dehydration, pain, diarrhea and fever, within 30 days. This process estimates the care provided to cancer patients, which is subsequently analyzed and improved with a focus on decreasing the number of unplanned hospitalizations. In addition to these measures, other strategies such as improving access to and providing coordinated care, developing standardized procedures for prompt identification and management of symptoms, and implementing urgent cancer care strategies, can significantly decrease not only hospitalization rates but also the duration of stay and the severity of these complications^32^.

### Limitations

In our study, we used E codes to identify patients who experienced hospitalizations for complications of systemic therapy. However, since the NIS is primarily an administrative database, E codes could be underrepresented because they are not obligatory for reimbursements. This may have resulted in an underestimation of the volume of hospitalizations for these conditions. Furthermore, the NIS does not have data on tumor staging or anti-cancer medications. Therefore, we could not stratify the complications based on disease severity or treatment regimen, thus limiting our findings. In addition, NIS erases all personal identifiers for data confidentiality. Thus, the same patient readmitted multiple times would have been considered as an independent new admission, leading to an overestimation of hospitalization rates. Despite these limitations, we decided to use NIS because data on hospitalization for complications of cancer treatment is sparse. In addition, we could accurately calculate national estimates due to the large sample size provided by the NIS, which is the largest, all payer in-patient database in the United States.

## Conclusions

The management of complications of cancer treatment pose a significant burden to hospitals. From 2005 to 2016, hospitalization rates for complications of systemic therapy increased by an annual rate of 8.1%, which is concerning, when compared to the fact that the overall hospitalization rate decreased by an annual rate of 0.5% during the same period. The most common complications of systemic therapy that required hospitalization were anemia, neutropenia, and sepsis. Among all complications, sepsis was associated with greatest length of stay, highest mortality, and highest expenditures. Improved strategies for identifying and managing these complications in the outpatient and emergency settings could significantly decrease these hospitalizations, the burden on the healthcare system, and subsequently improve the quality of life of cancer patients.

## Supplementary Information

Supplementary Information.

## References

[1] Cancer Statistics. https://www.cancer.gov/about-cancer/understanding/statistics.

[2] Cheng M, Jolly S, Quarshie WO, Kapadia N, Vigneau FD (2019). Modern radiation further improves survival in non-small cell lung cancer: an analysis of 288,670 patients. J. Cancer.

[3] Palumbo MO, Kavan P, Miller W (2013). Systemic cancer therapy: Achievements and challenges that lie ahead. Front. Pharmacol..

[4] Halpern MT, Yabroff KR (2008). Prevalence of outpatient cancer treatment in the United States: Estimates from the Medical Panel Expenditures Survey (MEPS). Cancer Invest..

[5] Du XL, Osborne C, Goodwin JS (2002). Population-based assessment of hospitalizations for toxicity from chemotherapy in older women with breast cancer. J. Clin. Oncol..

[6] Waddle MR, Chen RC, Arastu NH (2015). Unanticipated hospital admissions during or soon after radiation therapy: Incidence and predictive factors. Pract. Radiat. Oncol..

[7] Hassett MJ, Rao SR, Brozovic S (2011). Chemotherapy-related hospitalization among community cancer center patients. Oncologist.

[8] Brooks GA, Kansagra AJ, Rao SR, Weitzman JI, Linden EA, Jacobson JO (2015). A clinical prediction model to assess risk for chemotherapy-related hospitalization in patients initiating palliative chemotherapy. JAMA Oncol..

[9] Du XL, Chan W, Giordano S (2005). Variation in modes of chemotherapy administration for breast carcinoma and association with hospitalization for chemotherapy-related toxicity. Cancer.

[10] Ling DC, Kabolizadeh P, Heron DE (2015). Incidence of hospitalization in patients with head and neck cancer treated with intensity-modulated radiation therapy. Head Neck.

[11] Gangopadhyay A, Das J, Nath P, Maji T, Biswas J (2014). Incidence of hospitalization in patients receiving short course palliative cranial radiotherapy on outpatient basis in a limited resource setting: Experience from a regional cancer center in India. Rep. Pract. Oncol. Radiother..

[12] Pyenson BS FK. *Cancer Patients Receiving Chemotherapy: Opportunities for Better Management*. Milliman Inc. http://us.milliman.com/uploadedFiles/insight/research/health-rr/cancer-patients-receiving-chemotherapy.pdf.

[13] Overview of the National (Nationwide) Inpatient Sample (NIS). https://www.hcup-us.ahrq.gov/nisoverview.jsp. 10.7759/cureus.33111 PMC9884308 36721619

[14] Jairam V, Lee V, Park HS (2019). Treatment-related complications of systemic therapy and radiotherapy. JAMA Oncol..

[15] Khera R, Krumholz HM (2017). With great power comes great responsibility: Big data research from the national inpatient sample. Circulation.

[16] Houchens, R., Ross, D., Elixhauser, A. & Jiang, J. *Nationwide Inpatient Sample (NIS) Redesign Final Report. 2014. HCUP NIS Related Reports ONLINE*. (US Agency for Healthcare Research and Quality, 2017).

[17] Lang K, Marciniak MD, Faries D (2009). Trends and predictors of first-line chemotherapy use among elderly patients with advanced non-small cell lung cancer in the United States. Lung Cancer.

[18] Masters GA, Krilov L, Bailey HH (2015). Clinical cancer advances 2015: annual report on progress against cancer from the American Society of Clinical Oncology. J. Clin. Oncol..

[19] Bossi P, Botta L, Bironzo P (2019). Systematic review of adverse events reporting in clinical trials leading to approval of targeted therapy and immunotherapy. Future Oncol..

[20] Citrin DE (2017). Recent developments in radiotherapy. N. Engl. J. Med..

[21] McAlearney AS, Wellner J, Bickell NA (2013). Improving breast cancer care measurement and reporting in a complex, urban hospital setting. J. Healthc. Manag..

[22] Bejanyan N, Bolwell BJ, Lazaryan A (2012). Risk factors for 30-day hospital readmission following myeloablative allogeneic hematopoietic cell transplantation (allo-HCT). Biol. Blood Marrow Transplant..

[23] Hori Y, Sakamoto A, Goto T (2018). Analysis of Dietary intake during consecutive-Day chemotherapy for Bone and soft-Tissue sarcomas. Front. Nutr..

[24] Shadad AK, Sullivan FJ, Martin JD, Egan LJ (2013). Gastrointestinal radiation injury: symptoms, risk factors and mechanisms. World J. Gastroenterol..

[25] Luciano RL, Brewster UC (2014). Kidney involvement in leukemia and lymphoma. Adv. Chronic Kidney Dis..

[26] Goldhar HA, Yan AT, Ko DT (2016). The temporal risk of heart failure associated with adjuvant trastuzumab in breast cancer patients: A population study. J. Natl. Cancer Inst..

[27] Bouchlaka MN, Redelman D, Murphy WJ (2010). Immunotherapy following hematopoietic stem cell transplantation: Potential for synergistic effects. Immunotherapy.

[28] Mirabile A, Numico G, Russi EG (2015). Sepsis in head and neck cancer patients treated with chemotherapy and radiation: Literature review and consensus. Crit. Rev. Oncol..

[29] Danai PA, Moss M, Mannino DM, Martin GS (2006). The epidemiology of sepsis in patients with malignancy. Chest.

[30] Williams MD, Braun LA, Cooper LM (2004). Hospitalized cancer patients with severe sepsis: Analysis of incidence, mortality, and associated costs of care. Crit. Care.

[31] Akinosoglou K, Karkoulias K, Marangos M (2013). Infectious complications in patients with lung cancer. Eur. Rev. Med. Pharmacol. Sci..

[32] Rabello LS, Silva JR, Azevedo LC (2015). Clinical outcomes and microbiological characteristics of severe pneumonia in cancer patients: A prospective cohort study. PLoS ONE.

