# Supplementary material for: Hospitalization rates for complications due to systemic therapy in the United States
Source: Sci Rep. 2021 Apr 1;11:7385. doi: 10.1038/s41598-021-86911-x (PMC8016938; doi:10.1038/s41598-021-86911-x)
Supplement: Supplementary file 1 — Supplementary Information. [file 41598_2021_86911_MOESM1_ESM.docx]

**Supplementary Information**

**Hospitalization Rates for Complications due to Systemic Therapy in the Unites States**

Anshul Saxena PhD, Muni Rubens PhD, Venkataraghavan Ramamoorthy PhD, Raees Tonse MD, Emir Veledar PhD, Peter McGranaghan MS, Subrina Sundil, MD, Michael D Chuong, MD, Matthew D Hall, MD, Yazmin Odia MD, Minesh P Mehta MD, Rupesh Kotecha MD


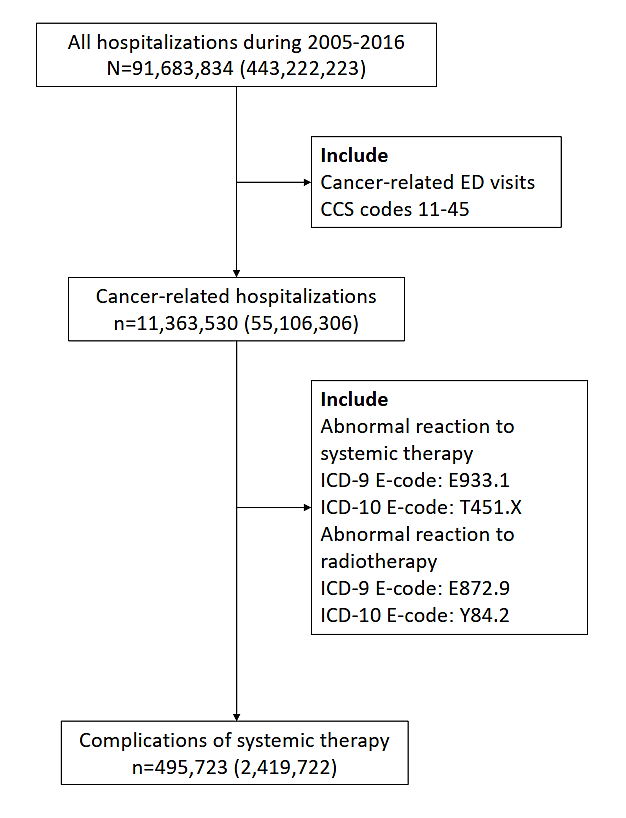
eFigure 1. Flow diagram showing the inclusion criteria used for the study

eTable 1: ICD and CCS codes used for defining complication of systemic therapy

| **Complications** | **ICD-9 Code** | **ICD-10 Code** | **CCS Code** |
| --- | --- | --- | --- |
| Neutropenia | 288.0x | D70.x | --- |
| Sepsis | --- | --- | 2 |
| Anemia | 284.x and 285.x | D61.x and D64.x | --- |
| Pneumonia | --- | --- | 122 |
| Nausea and vomiting | 536.2 | R11.10 | 250 |
| Dehydration | 276.51 | E86.0 | --- |
| Acute kidney injury | 584.x | N17.x | --- |
| Urinary tract infection | --- | --- | 159 |
| Fever of unknown origin | --- | --- | 246 |
| Congestive Heart Failure | --- | --- | 108 |

eTable 2. Demographic characteristics of hospitalizations for complications of systemic therapy

| **Variable** | **n (%, 95% CI)** |
| --- | --- |
| Age |  |
| 0-17 | 184100 (7.6%, 5.8%-7.3%) |
| 18-39 | 202489 (8.4%, 7.3%-7.9%) |
| 40-59 | 694529 (28.7%, 40.3%-41.2%) |
| ≥60 | 1338444 (55.3%, 44.2%-45.7%) |
| Missing | 160 (0.01%, 0.003%-0.008%) |
| Sex |  |
| Male | 1147478 (47.4%, 48.0%-48.5%) |
| Female | 1271231 (52.5%, 51.3%-51.8%) |
| Missing | 1013 (0.04%, 0.03%-0.05%) |
| Race |  |
| White | 1601226 (66.2%, 65.8%-67.9%) |
| Black | 228805 (9.5%, 9.1%-9.8%) |
| Hispanic | 210772 (8.7%, 7.7%-8.9%) |
| Asian or Pacific Islander | 65191 (2.7%, 2.4%-2.7%) |
| Native American | 9024 (0.37%, 0.32%-0.42%) |
| Other | 59403 (2.5%, 2.1%-2.6%) |
| Missing | 245301 (10.1%, 8.9%-11.1%) |
| Median household income |  |
| Quartile 1 | 557046 (23.0%, 22.5%-23.8%) |
| Quartile 2 | 589831 (24.4%, 23.8%-24.9%) |
| Quartile 3 | 611121 (25.3%, 24.7%-25.6%) |
| Quartile 4 | 610865 (25.2%, 24.2%-26.1%) |
| Missing | 50858 (2.1%, 1.9%-2.2%) |
| Primary payer |  |
| Medicare | 1064657 (44.0%, 45.5%-47.0%) |
| Medicaid | 321576 (13.3%, 12.2%-13.1%) |
| Private | 911991 (37.7%, 35.5%-36.7%) |
| Self-pay | 44995 (1.9%, 1.6%-1.9%) |
| No charge | 6390 (0.26%, 0.20%-0.32%) |
| Other | 65786 (2.7%, 2.4%-2.8%) |
| Missing | 4327 (0.18%, 0.12%-0.21%) |
| Region of hospital |  |
| Northeast | 440047 (18.2%, 17.2%-19.9%) |
| Midwest | 580966 (24.0%, 22.4%-25.4%) |
| South | 887656 (36.7%, 35.1%-38.4%) |
| West | 511053 (21.1%, 19.3%-22.0%) |
| Bed size of hospital |  |
| Small | 286660 (11.8%, 10.8%-12.6%) |
| Medium | 545629 (22.5%, 21.4%-23.7%) |
| Large | 1576496 (65.2%, 63.7%-66.6%) |
| Missing | 10936 (0.45%, 0.35%-0.64%) |
| Location/teaching status of hospital |  |
| Rural | 178430 (7.4%, 6.8%-8.2%) |
| Urban nonteaching | 686830 (28.4%, 27.8%-30.5%) |
| Urban teaching | 1543526 (63.8%, 61.2%-64.3%) |
| Missing | 10936 (0.45%, 0.35%-0.74%) |
| Mortality | 111655 (4.6%, 4.3%-4.7%) |
